# Proteomics analysis of lysine crotonylation and 2-hydroxyisobutyrylation reveals significant features of systemic lupus erythematosus

**DOI:** 10.1007/s10067-022-06254-4

**Published:** 2022-08-08

**Authors:** Ting Xie, Jingjing Dong, Xianqing Zhou, Donge Tang, Dandan Li, Jiejing Chen, Yumei Chen, Huixuan Xu, Wen Xue, Dongzhou Liu, Xiaoping Hong, Fang Tang, Lianghong Yin, Yong Dai

**Affiliations:** 1 Institute of Nephrology and Blood Purification, the First Affiliated Hospital of Jinan University, Guangzhou, 510632 Guangdong China; 2 Clinical Medical Research Center, the Second Clinical Medical College of Jinan University, Shenzhen People’s Hospital, Shenzhen, 518020 Guangdong China; 3 Department of Pathology, Guangxi Key Laboratory of Metabolic Diseases Research, No.924 Hospital of PLA Joint Logistic Support Force, Guilin, 541002 Guangxi China

**Keywords:** Systemic lupus erythematosus, Protein post-translational modification, Crotonylation, 2-Hydroxyisobutyrylation

## Abstract

**Introduction/objectives:**

To seek significant features of systemic lupus erythematosus (SLE) by utilizing bioinformatics analysis.

**Method:**

Liquid chromatography-tandem mass spectrometry (LC–MS/MS) was used to quantify lysine crotonylation (Kcr) and lysine 2-hydroxyisobutyrylation (Khib) in peripheral blood mononuclear cells (PBMCs) of systemic lupus erythematosus (SLE) patients and normal controls.

**Results:**

Seventy-six differentially modified proteins (DMPs) dually modified by Kcr and Khib were identified between SLE patients and healthy people. GO enrichment analysis prompted significant enrichment of seventy-six DMPs in MHC class II protein complex binding and leukocyte migration. KEGG pathways were enriched in antigen processing and presentation pathway and leukocyte transendothelial migration pathway. Six DMPs (CLTC, HSPA1B, HSPA8, HSP90AB1, HSPD1, and PDIA3) were identified in antigen processing and presentation pathway, of which HSPA8 was the core protein. Significant changes of Kcr and Khib in HSPA8 may increase ATP hydrolysis and promote antigen binding to MHC II molecule. In leukocyte transendothelial migration pathway, 7 DMPs (ACTN1, ACTN4, EZR, MSN, RAC1, RHOA, and VCL) were identified. MSN was the protein with the most modification sites in this pathway. In amino terminal ferm region of MSN, Kcr and Khib expression change may lead to the adhesion between leukocytes and endothelial cells, which was an important step of leukocyte migration.

**Conclusion:**

Kcr and Khib may promote the antigen presentation and jointly regulate the tissue damage mediated by leukocyte migration in SLE patients, which may play key roles in the pathogenesis of SLE probably.

**Table:** 

**Key Points** *• Antigen processing and presentation and leukocyte transendothelial migration may play key roles in the pathogenesis of SLE.*

**Supplementary Information:**

The online version contains supplementary material available at 10.1007/s10067-022-06254-4.

## Introduction

Systemic lupus erythematosus (SLE) is a chronic inflammatory disease related to autoimmunity and alternates between relapse and remission [1]. The etiology of SLE is unclear, which may be involved in genes, environment, and gender [2]. The covalent modifications occurred after RNA translated into protein are called PTMs, which diversify the proteome [3]. A large number of studies have shown the close relationship between PTMs and SLE [4–6]. Autoantigens in SLE are derived from apoptotic cells, and the process of apoptosis may induce many post-translational modifications [5].

Lysine crotonylation (Kcr) and lysine 2-hydroxyisobutyrylation (Khib) are two new PTMs identified in *Homo sapiens*, animals, and plants [7–9]. Kcr happens in the lysine in histones ε-amino upper and has been proved to be closely related to gene transcription and replication [7]. Kcr on non-histones may regulate processes including muscle contraction and protein synthesis [10]. Khib is related to the transcription of active genes in meiotic and posts meiotic cells, indicating its key role in regulating chromatin [8].

In living bodies, various PTMs do not exist in isolation. Cross-talk between different types of PTMs can produce a coordinated regulatory network to achieve various physiological functions. In the Protein Lysine Modification Database (Version 3.0) (http://plmd.biocuckoo.org/links.php), six histones were both modified with Kcr and Khib. Among these proteins, three histones were modified with the two PTMs simultaneously, including Histone H1.4, H2B type 1-B, and H3.1 [11]. Kcr caused a mass shift of + 68.0230 Da, while Khib caused a mass shift of + 86.0354 Da [7, 8]. Writer and eraser carry out the addition or removal modification of amino acid residue modification. As a transcriptional coactivator, P300 can catalyze many types of lysine acylation. P300 is a histone acetyltransferase and has the activities of histone crotonyltransferase and histone 2-hydroxyisobutyryltransferase [12–14]. Histone deacetylase (HDAC) could remove Kcr and Khib in vivo [15, 16], while suberoylanilide hydroxamic acid (SAHA), a HDAC inhibitor, was highly correlated to the change of Kcr and Khib modification in lung cancer cells [17]. Increasing evidence has emerged to support the close relationship between Kcr and Khib.

Therefore, we hypothesize that Kcr and Khib are related to the pathogenesis of SLE that is a classic autoimmune disease. This study intends to find the features of overlapping Kcr and Khib modification of PBMCs in patients with SLE using LC–MS/MS-based proteomic analysis.

## Methods

### Sample preparation

Following the principle of informed consent and under the direction of a protocol approved by the No. 924 Hospital of PLA Joint Logistic Support Force Ethics Committee, 3.5-ml peripheral blood samples from 11 SLE patients and 36 healthy people were gathered for Kcr proteomics analysis. Moreover, 3.5-ml peripheral blood samples from 8 SLE patients and 20 healthy people were collected for Khib proteomics analysis. (Clinical information of SLE patients was shown in Table S1 and Table S2 of the Supplementary Information.) PBMCs were isolated by density gradient centrifugation. The BCA kit determined the protein supernatant concentration. Trypsin was added to the protein solution, which hydrolyzed protein into peptides. Peptides were labeled with TMT reagent. Peptides were dissolved in buffer solution, transferred to crotonylation or 2-hydroxyisobutyrylation washed in advance for the enrichment, and eluted after overnight incubation. Then, peptides were analyzed by mass spectrometry. The mass spectrometry data were retrieved using Maxquant (v1.5.2.8) to match the corresponding proteins. (More detailed trial procedures are shown in the Supplementary Information.)

### Bioinformatics analysis

Proteins with a change in differential folding value greater than 1.5 times or less than 1/1.5 are defined as DMPs. DMPs dually modified by Kcr and Khib were screened by Venn diagram (https://bioinfogp.cnb.csic.es/tools/venny/index.html), and WOLF-PSORT was used to classify subcellular localization. The function and features of DMPs dually modified by Kcr and Khib were annotated by Gene Ontology (GO) enrichment analysis. The interaction was annotated by the Kyoto Encyclopedia of Genes and Genomes (KEGG) enrichment analysis. STRING and Cytoscape v3.9.0 were used to construct PPI networks. Core genes were identified by the CytoHubba plugin.

## Result

### Protein identification

**Fig. 1:**
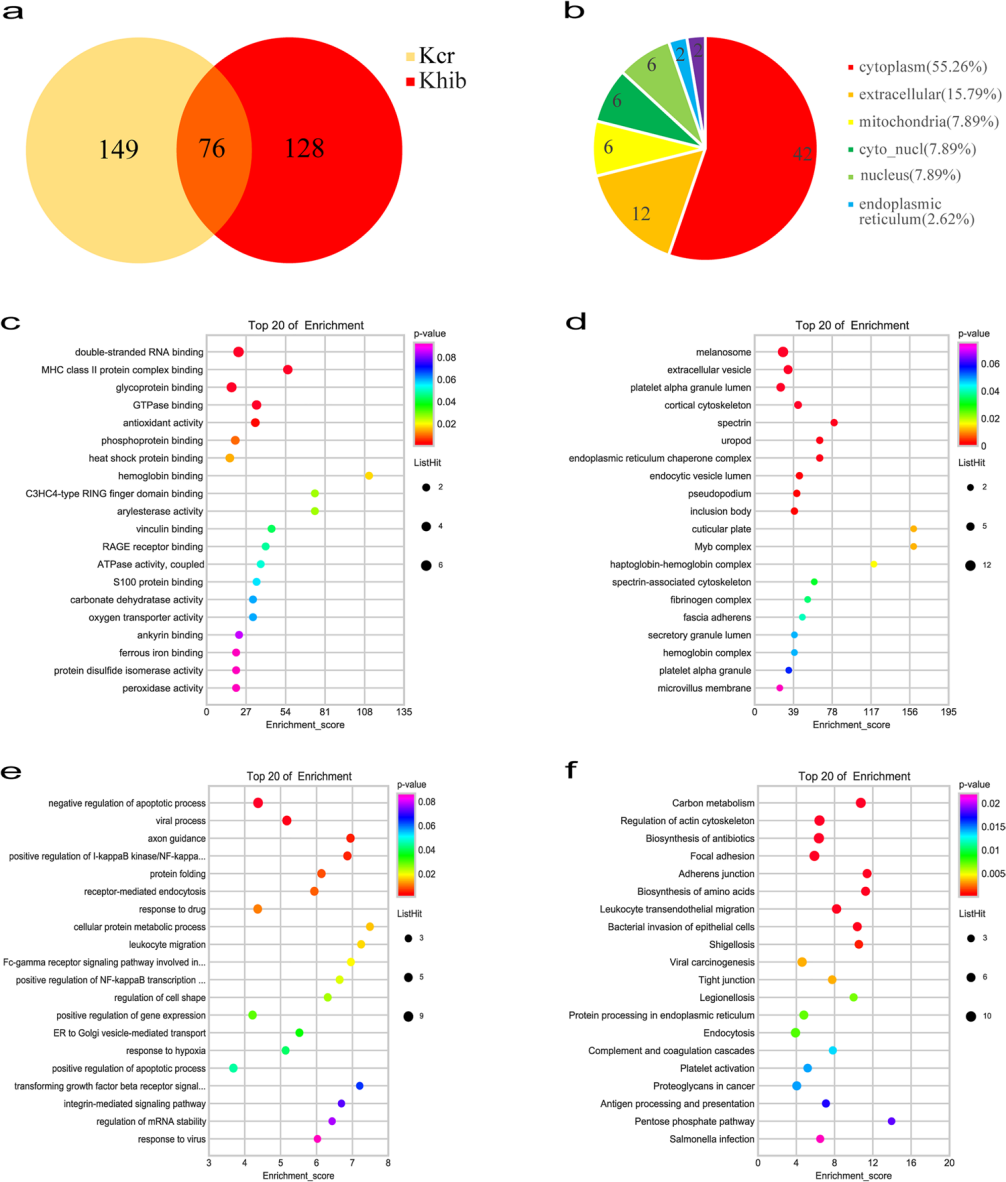
Differentially quantified proteins of lysine crotonylation and 2-hydroxyisobutyrylation between SLE patients and healthy people. (**a**) Venn diagram showing the DMPs. (**b**) Subcellular location of the DMPs. (**c**–**f**) GO enrichment analysis and KEGG pathway enrichment analysis of the DMPs. (**c**) Cellular component in the GO enrichment analysis. (**d**) Molecular function in the GO enrichment analysis. (**e**) Biological process in the GO enrichment analysis. (**f**) KEGG analysis of the DMPs

A total of 1209 crotonylation sites on 377 proteins were identified in the SLE group and normal control (NC) group. Among them, 1109 sites on 347 proteins had quantitative information. In addition, 3684 sites on 1036 proteins were identified by 2-hydroxyisobutyrylation proteomics analysis in SLE patients and normal controls. Among them, 1109 sites on 347 proteins had quantitative information. Taking the difference fold value change more than 1.5 times as a significant upregulation and less than 1/1.5 as a significant downregulation criterion, 761 Kcr sites on 225 proteins and 407 Khib sites on 258 proteins were differentially expressed between the SLE group and NC group. In the Venn diagram (Fig. 1a), 76 DMPs were co-modified by Kcr and Khib. The 76 DMPs dually modified by Kcr and Khib accounted for 33.7% in the total Kcr proteins and 29.4% in Khib proteins.

### The features of DMPs dually modified by Kcr and Khib

The subcellular localization of DMPs dually modified by Kcr and Khib was distributed in the cytoplasm (53.95%), extracellular (15.79%), mitochondria (9.21%), cyto_nucl (7.89%), nucleus (7.89%), endoplasmic reticulum (2.62%), and plasma membrane (2.62%) (Fig. 1b). Then, we used the GO analysis to investigate the functional category distribution of the 76 overlapping DMPs of dual Kcr and Khib. These proteins were separated into three groups: cellular component (31.09%), molecular function (20.17%), and biological process (48.74%).

### Functional enrichment of DMPs dually modified by Kcr and Khib

We used GO enrichment analysis to understand the functions of 76 DMPs dually modified by Kcr and Khib in SLE patients. In cellular component, these proteins were highly enriched in cuticular plate, Myb complex, haptoglobin-hemoglobin complex, spectrin, and uropod (Fig. 1c). In the molecular functions, these proteins were closely related with hemoglobin binding, arylesterase activity, C3HC4-type RING finger domain binding, MHC class II protein complex binding, and heat shock protein binding (Fig. 1d). In the biological processes, the cellular protein metabolic process, leukocyte migration, and transforming growth factor-beta receptor signaling pathway involved in phagocytosis were highly enriched (Fig. 1e).

KEGG pathway enrichment analysis found that 76 DMPs dually modified by Kcr and Khib were enriched in carbon metabolism, regulation of actin cytoskeleton, antigen processing and presentation, and leukocyte transendothelial migration (Fig. 1f).

### Characteristic of antigen processing and presentation pathway

**Fig. 2:**
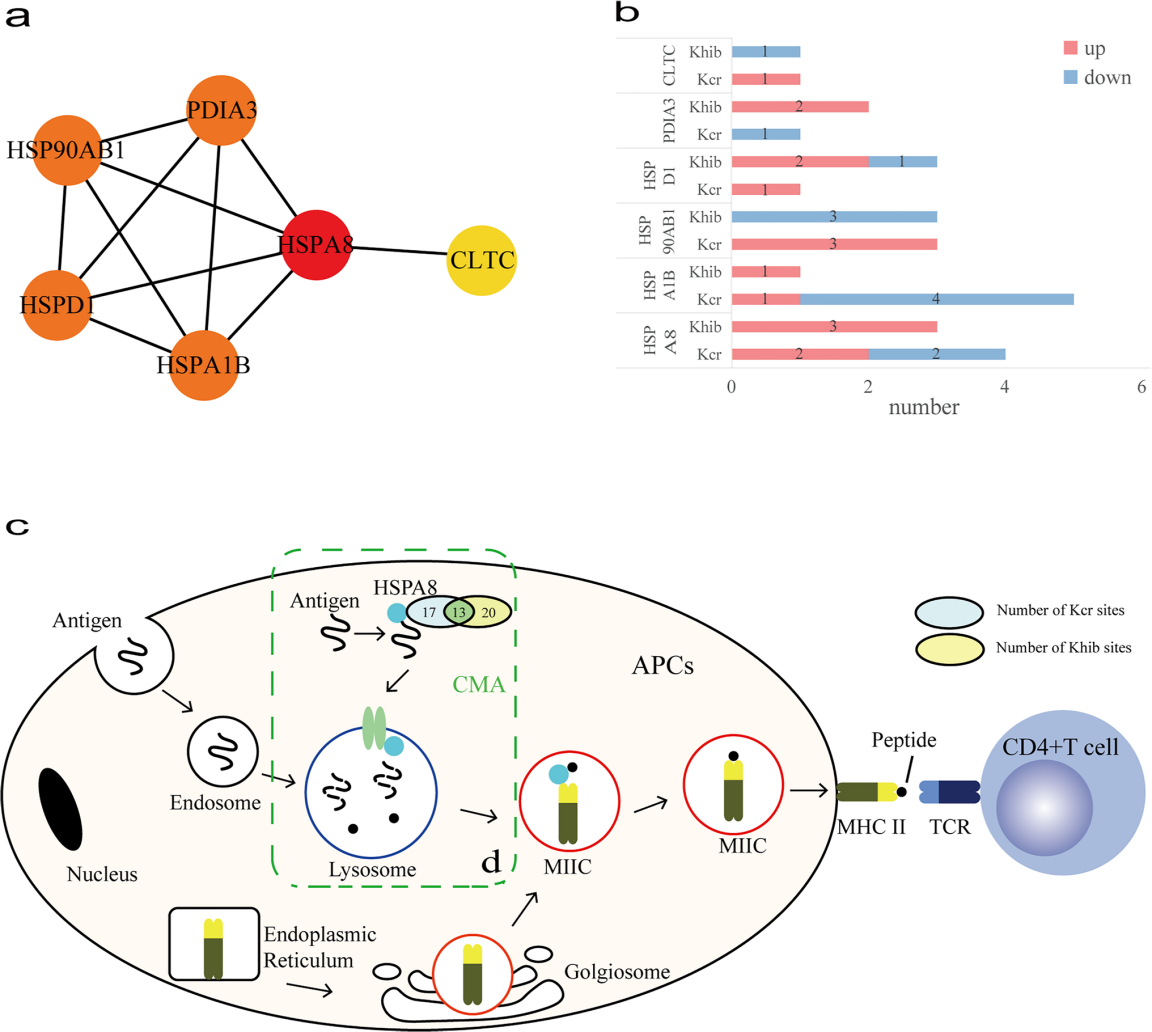
DMPs dually modified by Ker and Khib in the antigen processing and presentation. (**a**) PPI network of DMPs enriched antigen processing and presentation. (**b**) Numbers of modified sites of Ker and Khib in antigen processing and presentation. (**c**) KEGG pathway of antigen processing and presentation. (**d**) CMA, chaperone-mediated autophagy

The process of antigen processing and presentation was a highly enriched pathway in KEGG analysis and is closely associated with the development of SLE. In the PPI network, it contained six nodes, including CLTC, HSPA1B, HSPA8, HSP90AB1, HSPD1, and PDIA3, and 11 interactions (Fig. 2a). According to CytoHubba, HSPA8 was the top hub gene in this network. HSPA8 was the most abundant dually modified protein with 17 Kcr sites and 20 Khib sites. Especially, there were thirteen sites (K71, K108, K128, K159, K246, K319, K328, K451, K500, K526, K531, and K589) co-modified by both Kcr and Khib in HSPA8 protein. In addition, the Kcr of four sites (K187, K451, K512, and K531) and the Khib of three sites (K159, K246, and K357) were different expression (Table S3, Supplementary Information). The differential modification sites of other five proteins are shown in the Supplementary Information (Table S4–8).

### Characteristic of leukocyte transendothelial migration pathway

**Fig. 3:**
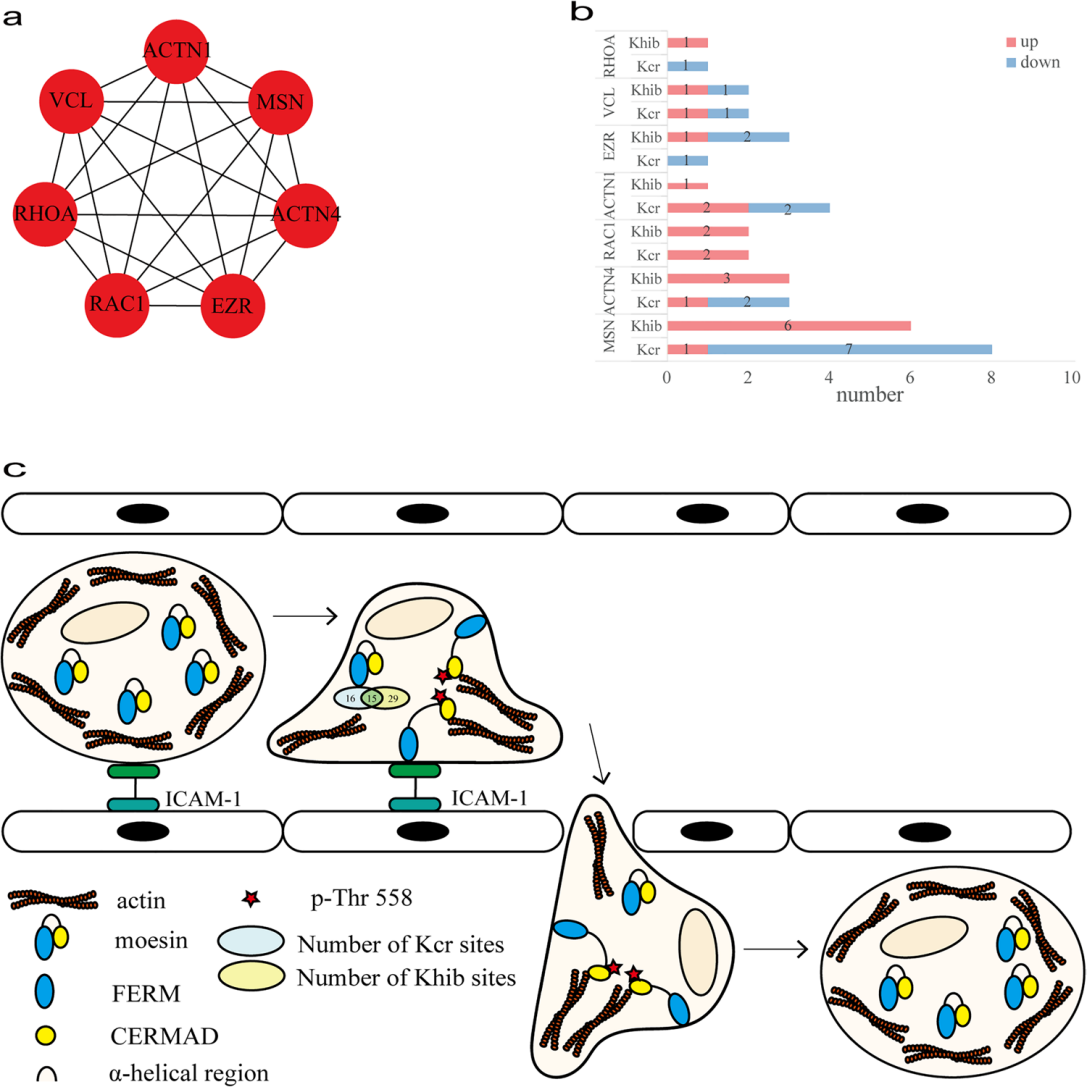
DMPs and OMS of dual Ker and Khib in the leukocyte transendothelial migration. (**a**) PPI network of DMPs enriched leukocyte transendothelial migration. (**b**) Numbers of modified sites of Ker and Khib in the leukocyte transendothelial migration. (**c**) KEGG pathway of the leukocyte transendothelial migration

The process of leukocyte transendothelial migration highly enriched in KEGG analysis was paid attention to for the close relationship with autoimmune disease. The PPI network contained seven nodes, including ACTN1, ACTN4, EZR, MSN, RAC1, RHOA, and VCL, and 21 interactions (Fig. 3a). Moesin encoded by MSN was involved in the leukocyte transendothelial migration process. Moesin was the most abundant dually modified protein with 16 Kcr sites and 29 Khib sites. There were 15 sites (K64, K72, K79, K162, K165, K237, K253, K316, K327, K344, K352, K388, K400, K458, and K514) co-modified by both Kcr and Khib. Among these sites, eight Kcr sites (K79, K316, K335, K352, K388, K400, K458, and K514) and 6 Khib sites (K72, K79, K83, K151, K263, and K523) were differentially modified in moesin protein between the SLE group and normal control group (Table S9, Supplementary Information). The differential modification sites of other six proteins are shown in the Supplementary Information (Table S10–15).

## Discussion

Recently, more and more PTMs have been proved to be related to SLE. Our study explored the features of Kcr and Khib modification in patients who diagnosed SLE. In this study, by using LC–MS/MS-based proteomic analysis, we identified 76 DMPs dually modified by Kcr and Khib between SLE patients and healthy people. In the GO enrichment analysis, 76 DMPs were enriched in the process of MHC class II protein complex binding and leukocyte migration. The result of KEGG analysis was enriched in antigen processing and presentation pathway and leukocyte transendothelial migration pathway. Then, we further focus on the above two pathways which have the potential to be the pathogenesis of SLE.

It is generally believed that SLE, a chronic and complex disease, is characterized by the production of many autoantibodies, which combine with autoantigens to form immune complexes and then deposit in local tissues, causing an inflammatory reaction and tissue damage [18]. The autoantigens in SLE mainly come from apoptotic cells [5]. The autoantigens are usually promoted to CD4 + T lymphocytes by antigen presenting cells through MHC II molecules [19, 20]. With the help of HSC70 (coded by HSPA8), translocation of cytoplasmic antigens into endosomes/lysosomes is mediated by autophagy, especially chaperone-mediated autophagy (CMA) [21, 22]. In addition, QKRAA amino acid motifs and RRRAA amino acid motifs of HLA-DR (a kind of human MHC II molecule) mediate the binding of HLA-DR to HSPA8 [23]. In our study, HSPA8 was upregulated in SLE patients. In molecular functions by GO analysis, HSPA8 was enriched in the process of MHC class II protein complex binding. And HSPA8 was significantly enriched in antigen processing and presentation pathway in KEGG analysis. Abundant Kcr and Khib modification sites were identified in HSPA8 in SLE patients. Our study found that HSPA8 had 13 sites co-modified by both Kcr and Khib, in which the modification of K451 was significantly downregulated, and the modification of K159, K246, and K531 was significantly upregulated. HSPA8 contains an N-terminal domain (NBD, the ATPase domain) and a C-terminal domain (SBD, binding peptide/protein substrate) [24]. ATP was hydrolyzed to ADP by NBD of HSPA8 while the SBD structure opened, and the peptide binding to SBD would increase the ATP hydrolysis rate [24]. K159 and K246 located in NBD and K531 located in SBD were significantly upregulated. HSPA8 protects antigenic peptides from decomposition in lysosomes and rapidly transfers them to empty MHC II molecules using the energy of ATP hydrolysis [25]. It is speculated that the significant changes of Kcr and Khib in NBD and SBD of HSPA8 may increase ATP hydrolysis and promote the binding of antigen to MHC II molecule, which may be related to the pathogenesis of SLE.

The presence of immune complexes triggers the activation of the complement system, resulting in inflammation and complement protein consumption [26]. Inflammatory mediators have leukocyte chemotaxis, which makes leukocytes gather at the site of immune complex deposition [27, 28]. The phenotype of SLE neutrophils was different from that of healthy individuals, and neutrophils were activated and easy to aggregate [29]. Neutrophils and macrophages release lysosomal enzymes causing damage to the surrounding tissue [30]. Leukocyte migration is an important and complex process requiring many adhesion molecules, chemokines, and receptors. The main processes are rolling adhesion mediated by selectin, activation triggered by chemokine signal, and firm adhesion mediated by integrin [31]. After firm binding, through signal transduction, with the participation of actin cytoskeleton contraction, leukocytes move forward through the protrusion and retraction of the tail foot, and leukocytes can migrate through the open endothelial cell channel [32, 33]. In our study, seven DMPs were associated with leukocyte transendothelial migration pathway. Especially, MSN, encoding moesin, was involved in this biological process of leukocyte migration by GO enrichment analysis. Moesin is located in the cell membrane and is a bridge connecting the actin cytoskeleton and transmembrane proteins, playing a crucial role in maintaining both the tissue cytoskeleton and cell morphology and coordinating intercellular signaling [34, 35]. Endothelial cells bind to leukocytes through intercellular adhesion molecule (ICAM-1) and ERM to activate the RhoA-ROCK-MLC signaling pathway, resulting in the contraction of the actin cytoskeleton [36, 37]. In phenotypes of moesin knockout (MSN − /y) mice, neutrophil migration and chemotaxis are reduced, so the role of neutrophil-mediated microbial killing and inflammation is weakened [38]. We detected that moesin was the most abundant DMP in our study. K79 on moesin was co-modified by Kcr and Khib. The Kcr modification level of K79 was significantly downregulated, while the Khib was significantly upregulated. Moesin consists of three parts: (1) the amino terminal ferm region (FERM) that can bind to the cell membrane; (2) the α-helical region; (3) the carboxyl terminal cermad region (CERMAD) that can bind to actin constitute [39]. In moesin sleep, the self-folding of the α-helical region masks the tight binding of FERM to CERMAD [39]. CERMAD was isolated from FERM by phosphorylation of Thr558 and exposed to actin binding sites to activate moesin [40, 41]. K514 was adjacent to Thr558 and was located at CERMAD. Kcr and Khib can also neutralize the positive charge of amino group as phosphorylation [42]. Therefore, we speculate that Kcr and Khib in K514 were also involved in the activation of moesin. In addition, serine phosphorylation of cell membrane molecules binds to the FERM domain by reducing the net positive charge of the FERM binding motif [35]. K79 was located in FERM, so we speculate that Kcr and Khib of FERM may neutralize the positive charge of the amino group to promote the binding of cell membrane molecules. Therefore, it is speculated that a dynamic relationship between crotonylation and 2-hydroxyisobutyrylation would jointly regulate leukocyte migration-mediated tissue damage.

Certainly, this study also has shortcomings. Firstly, this study focused on the overall characteristics of Kcr and Khib in SLE patients without considering the subgroups of SLE patients according to disease activity and severity and the organ involved. Secondly, the number of SLE patients in this study was small. Overall, this work provides a new direction on the possible implication of Kcr and Khib in the pathogenesis of patients with SLE.

## Conclusion

In conclusion, our quantitative proteomic analysis investigated double modified proteins with Kcr and Khib in PBMCs from SLE patients and healthy people. Our study showed that SLE is a disease related to various proteins and signaling pathways, especially antigen processing and presentation and leukocyte transendothelial migration, which provides a new understanding of the pathogenesis of SLE.

## Supplementary Information

Below is the link to the electronic supplementary material.

Supplementary file1 (DOCX 62 kb)

## Data Availability

The datasets generated and analyzed during the current study are available in the PRIDE repository. PXD012966, Username: reviewer31298@ebi.ac.uk, Password: W31S9RX. PXD015351, Username: reviewer73483@ebi.ac.uk, Password: 2qd6MgH1.
