# Supplementary material for: Proteomics analysis of lysine crotonylation and 2-hydroxyisobutyrylation reveals significant features of systemic lupus erythematosus
Source: Clin Rheumatol. 2022 Aug 8;41(12):3851–8. doi: 10.1007/s10067-022-06254-4 (PMC9652266; doi:10.1007/s10067-022-06254-4)
Supplement: Supplementary file 1 — Supplementary file1 (DOCX 62 kb) [file 10067_2022_6254_MOESM1_ESM.docx]

**Supplementary Information**

Proteomics analysis of lysine crotonylation and 2-hydroxyisobutyrylation reveals significant features of systemic lupus erythematosus

Ting Xie1, Jingjing Dong1,2, Xianqing Zhou3, Donge Tang2, Dandan Li1, Jiejing Chen3, Yumei Chen2, Huixuan Xu2, Wen Xue3,Dongzhou Liu2, Xiaoping Hong2, Fang Tang3*, Lianghong Yin1*, Yong Dai2*,

1 Institute of Nephrology and Blood Purification, the First Affiliated Hospital of Jinan University, Jinan University, Guangzhou, Guangdong 510632, China.

2 Clinical Medical Research Center, the Second Clinical Medical College of Jinan University, Shenzhen People’s Hospital, Jinan University, Shenzhen Guangdong 518020, China.

3 Department of Pathology, No.924 Hosptital of PLA Joint Logistic Support Force, Guangxi Key laboratory of Metabolic Diseases Research, Guilin Guangxi 541002, China.

***Corresponding authors:**

Prof. Fang Tang

Department of Pathology, No.924 Hosptital of PLA Joint Logistic Support Force, Guangxi Key laboratory of Metabolic Diseases Research, GuiLin Guangxi 541002, China, E-mail address: fangtang@163.com

Prof. Lianghong Yin

Institute of Nephrology and Blood Purification, the First Affiliated Hospital of Jinan University, Jinan University, Guangzhou, Guangdong 510632, China, Tel: +86 020-38688449, E-mail address: yin-yun@126.com

Prof. Yong Dai

The Second Clinical Medical College of Jinan University, Shenzhen People’s Hospital, Shenzhen Guangdong 518020, China, Tel: +86 0755-22942780, E-mail address: daiyong22@aliyun.com

**Funding Information:**

the Fund of Guangxi Key laboratory of Metabolic Diseases Research (grant number No.20-065-76)

The authors have no conflicts of interest to disclose.

The datasets generated and analyzed during the current study are available in the PRIDE repository. PXD012966, Username: reviewer31298@ebi.ac.uk, Password: W31S9RX.

PXD015351, Username: reviewer73483@ebi.ac.uk, Password: 2qd6MgH1.

**Materials and Methods**

1. Patients and healthy people

Following the principle of informed consent and under the direction of a protocol approved by the Guangxi Key Laboratory of Metabolic Diseases Research Ethics Committee, we gathered 3.5 ml peripheral blood samples from 11 SLE patients and 36 healthy people for researching crotonylation. Moreover, we gathered 3.5 ml peripheral blood samples from 8 SLE patients and 20 healthy people for researching 2-hydroxyisobutylation. PBMCs were separated from peripheral blood samples by density gradient centrifugation using Ficoll-Hypaque and then stored at -80℃.

2. Protein Extraction

Take out the sample from -80 °C and add 4 times the volume of cracking buffer (8 M urea, 1% protease inhibitor). In addition, in the PTM experiment, other inhibitors (such as 50 mM NAM and 3 μM TSA for acetylation) were added to the cracking buffer. Then, the supernatant was obtained by centrifugation at 12,000g for 10 min at 4 °C, and the protein concentration was determined with the BCA kit according to the manufacturer's instructions.

3. Trypsin Digestion

Dithiothreitol was added to the protein solution to make the final concentration of 5 mM and reduced at 56 °C for 30 min. After that, iodoacetamide was added to make the final concentration of 11 mM and incubated at room temperature in the dark for 15 min. Finally, the urea concentration of the sample was diluted to less than 2M by adding 100 mM TEAB. Trypsin was added in a mass ratio of 1:50 (trypsin: protein), and enzymolysis was performed overnight at 37 ° C. Then add trypsin in the mass ratio of 1:100 (trypsin: protein) and continue enzymatic hydrolysis for 4 hours.

4. TMT/iTRAQ marking

The peptides hydrolyzed by trypsin were desalted with strata x C18 (phenomenex) and vacuum freeze-dried. The peptide segment was dissolved with 0.5 M TEAB and the labeled peptide segment was described according to the TMT/iTRAQ kit operation. The simple operations were as follows: the labeled reagent was thawed and dissolved in acetonitrile, mixed with the peptide, and incubated at room temperature for 2 hours. The labeled peptides were mixed and desalted and vacuum freeze-dried.

5. Affinity Enrichment

Tryptic peptides were dissolved in IP buffer solution (100 mM NaCl, 1 mm EDTA, 50 mm Tris HCl, 0.5% NP-40, pH 8.0). The supernatant was transferred to crotonoyl antibody beads (PTM bio) washed in advance and placed on a rotating shaking table at 4 °C, gently shaken, and incubated overnight. Beads were washed four times with IP buffer solution and twice with deionized water after incubation. Tryptic peptides were eluted from the beads with 0.1% trifluoroacetic acid eluent. Finally, tryptic peptides were desalted according to the instructions of C18 Zip Tips and vacuum freeze-dried for LC-MS/MS analysis.

6. LC-MS/MS Analysis

Tryptic peptides were dissolved in 0.1% formic acid (solvent A) and directly loaded onto a homemade reversed-phase analytical column (15 cm long, 75 cm long μM inner diameter). The gradient included an increase from 6% to 23% of solvent B (0.1% formic acid in 98% acetonitrile) in 26 minutes, an increase from 23% to 35% in 8 minutes, an increase to 80% in 3 minutes, and then maintained at 80% in the last 3 minutes. These processes were carried out at a constant flow rate of 400 NL /min on the EASY-nLC 1000 UPLC system. After separation by UPLC system, peptides were injected into NSI ion source for ionization, and then analyzed by Q ExactiveTM Plus (Thermo) mass spectrometry. The ion source voltage was set at 2.0 kV, and the peptide parent ions and their secondary fragments were detected and analyzed by high-resolution Orbitrap. The scanning range of primary mass spectrometry was set to 350-1800 m/z, and the scanning resolution was set to 70000. By setting the normalized collision energy to 28, peptides for MS/MS were selected, and fragments were detected in Orbitrap at a resolution of 17500. Data-related procedures alternately performed between 1 MS/MS scan and 20 MS/MS scan. The standard dynamic exclusion duration was 15.0 s. The automatic gain control parameter is set to 5E4. The fixed first mass was set as 100 m/z.

7. Database Search

The resulting MS/MS data were analyzed by a Maxquant search engine (v.1.5.2.8). Connecting the KCR database or khib database with the reverse decoy database was used for tandem MS data search. Trypsin/P was designated as a cleavage enzyme that allowed up to 4 missing cleavages. In the first search, the mass tolerance was set to 20 ppm for precursor ions while 5 ppm in the main search. For fragment ionsand, the mass tolerance was set to 0.02 Da. Carbamidomethyl modification was designated as a fixed modification on Cys. Kcr, Khib, and oxidation on Met were designated as variable modifications. The false discovery rate was adjusted to below 1%, and the minimum score for modified peptides was set to exceed 40.

8. Bioinformatic Methods

Proteins with a change in differential folding value greater than 1.5 times or less than 1 / 1.5 are defined as differentially modified proteins (DMPs). Proteins modified by crotonylation and 2-hydroxyisobutyrylation were screened by Venn Diagram 2.1.0 (https://bioinfogp.cnb.csic.es/tools/venny/index.html), and WOLF-PSORT was used to predict and classify subcellular localization. The function and features of DMPs were annotated by Gene Ontology (GO) enrichment analysis, and the interaction was annotated by Kyoto Encyclopedia of genes and genes (KEGG) enrichment analysis. STRING and Cytoscape v3.9.0 were used to construct PPI networks of DMPs involved in antigen processing and presentation and leukocyte transendothelial migration pathways and identified core genes by the CytoHubba plugin.

| No. | Contents | Page |
| --- | --- | --- |
| Table S1 | Clinical information of SLE patients identified Kcr | VI |
| Table S2 | Clinical information of SLE patients identified Khib | VII |
| Table S3 | Kcr and Khib sites on HSPA8 in this study | VII |
| Table S4 | Kcr and Khib sites on HSPA1B in this study | VIII |
| Table S5 | Kcr and Khib sites on HSP90AB1 in this study | VIII |
| Table S6 | Kcr and Khib sites on HSPD1 in this study | IX |
| Table S7 | Kcr and Khib sites on PDIA3 in this study | IX |
| Table S8 | Kcr and Khib sites on CLTC in this study | IX |
| Table S9 | Kcr and Khib sites on MSN in this study | X |
| Table S10 | Kcr and Khib sites on ACTN4 in this study | XI |
| Table S11 | Kcr and Khib sites on ACTN1 in this study | XI |
| Table S12 | Kcr and Khib sites on RAC1 in this study | XII |
| Table S13 | Kcr and Khib sites on EZR in this study | XII |
| Table S14 | Kcr and Khib sites on VCL in this study | XII |
| Table S15 | Kcr and Khib sites on RHOA in this study | XIII |

| Table S1 Clinical information of SLE patients identified Kcr | | | | | | | | | | | | | | | | | | |
| --- | --- | --- | --- | --- | --- | --- | --- | --- | --- | --- | --- | --- | --- | --- | --- | --- | --- | --- |
| NO | Gender | Age | WBC | RBC | HGB | PLT | MPV | Urea | Cr | UA | IgG | IgA | IgM | C3 | C4 | CRP | dsDNA | ANA |
| P1 | female | 14 | 10.63 | 3.27 | 93 | 87 | 12.8 | 3.8 | 51 | 269 | 12.31 | 1.96 | 3.72 | 0.345 | 0.02 | 43.81 | 1214.3 | 1:3200  Positive |
| P2 | female | 36 | 4.55 | 4.45 | 127 | 98 | 9.6 | 4.5 | 93 | 349 | 21.23 | 1.95 | 0.48 | 0.44 | 0.11 | <5.0 | 381.2 | 1:3200  Positive |
| P3 | female | 28 | 3.48 | 2.48 | 75 | 218 | 10.7 | 24.3 | 699 | 624 | 10.9 | 2.56 | 0.67 | 0.36 | 0.13 | <5.0 | 926.5 | 1:1000  Positive |
| P4 | female | 15 | 10.43 | 3.79 | 103 | 263 | 10.4 | 11.1 | 109 | 810 | 341 | 1.75 | 0.54 | 0.43 | 0.06 | <5.0 | 664.6 | 1:1000  Positive |
| P5 | female | 9 | 13.67 | 2.05 | 117 | 306 | 10.4 | 2.8 | 53 | 395 | 13.49 | 3.51 | 1.61 | 0.72 | 0.06 | <5.0 | 960.6 | 1:3200  Positive |
| P6 | female | 30 | 3.35 | 2.82 | 72 | 135 | 8.8 | 10.5 | 203 | 322 | 12.26 | 1.86 | 0.87 | 0.19 | 0.06 | <5.0 | 487.9 | 1:3200  Positive |
| P7 | female | 47 | 4.71 | 2.51 | 77 | 90 | 8.9 | 14.3 | 294 | 544 | 8.8 | 0.29 | 0.34 | 0.81 | 0.31 | 48.42 | 6.6 | 1:320  Positive |
| P8 | female | 34 | 8.96 | 4.26 | 123 | 226 | 8.3 | 4.1 | 102 | 233 | 10.07 | 2.36 | 1.02 | 0.89 | 0.2 | <5.0 | 27.1 | 1:3200  Positive |
| P9 | female | 53 | 10.69 | 1.89 | 51 | 113 | 9 | 20.1 | 341 | 496 | 8.48 | 1.17 | 0.6 | 0.36 | 0.04 | 5.17 | 5.7 | 1:10000  Positive |
| P10 | female | 45 | 10.44 | 2.14 | 94 | 142 | 9.9 | 8.2 | 80 | 496 | 12.9 | 3.4 | 0.51 | 1.26 | 0.21 | <5.0 | 98.1 | 1:1000 Positive |
| P11 | female | 35 | 8.34 | 4.61 | 132 | 204 | 7.5 | 4.9 | 72 | 333 | / | / | / | / | / | <5.0 | 139 | 1:10000  Positive |

| Table S2 Clinical information of SLE patients identified Khib | | | | | | | | | | | | | | | | | |
| --- | --- | --- | --- | --- | --- | --- | --- | --- | --- | --- | --- | --- | --- | --- | --- | --- | --- |
| NO | Gender | Age | WBC | RBC | HGB | PLT | MPV | Urea | Cr | UA | IgG | IgA | IgM | C3 | C4 | CRP | ANA |
| P1 | male | 48 | 5.82 | 4.55 | 124 | 136 | 11.2 | 6.2 | 73 | 228 | 16.92 | 4.30 | 1.28 | 0.26 | 0.01 | <5.0 | 1:10000  Positive |
| P2 | female | 55 | 11.2 | 3.42 | 108 | 166 | 8.6 | 9.9 | 139 | 217 | 5.48 | 1.52 | 0.17 | 1.08 | 0.24 | 8.77 | 1:3200  Positive |
| P3 | female | 17 | 7.64 | 4.92 | 135 | 371 | 11.0 | 2.5 | 60 | 336 | 11.38 | 1.51 | 0.89 | 1.18 | 0.12 | 42.34 | 1:3200  Positive |
| P4 | male | 80 | 6.83 | 2.44 | 79 | 269 | 7.1 | 22.3 | 729 | 216 | 19.1 | 0.83 | 0.40 | 0.90 | 0.13 | <5.0 | 1:1000  Positive |
| P5 | female | 60 | 6.37 | 3.05 | 88 | 124 | 11.0 | 10.2 | 63 | 231 | 11.11 | 1.90 | 0.75 | 0.75 | 0.01 | / | / |
| P6 | female | 57 | 8.92 | 3.52 | 102 | 301 | / | / | / | / | 17.64 | 3.86 | 0.86 | 0.59 | 0.09 | 13.38 | 1:10000  Positive |
| P7 | female | 28 | 9.14 | 3.58 | 109 | 117 | 8.4 | 11.9 | 146 | 494 | / | / | / | / | / | <5.0 | 1:100  Positive |
| P8 | female | 25 | 6.49 | 2.13 | 61 | 115 | 7.7 | 24.8 | 436 | 257 | / | / | / | / | / | <5.0 | 1:1000  Positive |

| Table S3 Kcr and Khib sites on HSPA8 in this study | | | | | |
| --- | --- | --- | --- | --- | --- |
| Protein accession | Position | Amino  acid | Gene  name | Kcr  regulation | Khib  regulation |
| P11142 | 159 | K | HSPA8 | - | up |
| P11142 | 187 | K | HSPA8 | up | - |
| P11142 | 246 | K | HSPA8 | - | up |
| P11142 | 357 | K | HSPA8 | - | up |
| P11142 | 451 | K | HSPA8 | down | - |
| P11142 | 512 | K | HSPA8 | down | - |
| P11142 | 531 | K | HSPA8 | up | - |

| Table S4 Kcr and Khib sites on HSPA1B in this study | | | | | |
| --- | --- | --- | --- | --- | --- |
| Protein accession | Position | Amino  acid | Gene  name | Kcr  regulation | Khib  regulation |
| P0DMV9 | 71 | K | HSPA1B | - | up |
| P0DMV9 | 257 | K | HSPA1B | up | - |
| P0DMV9 | 500 | K | HSPA1B | down | - |
| P0DMV9 | 507 | K | HSPA1B | down | - |
| P0DMV9 | 512 | K | HSPA1B | down | - |
| P0DMV9 | 526 | K | HSPA1B | down | - |

| Table S5 Kcr and Khib sites on HSP90AB1 in this study | | | | | |
| --- | --- | --- | --- | --- | --- |
| Protein accession | Position | Amino  acid | Gene  name | Kcr  regulation | Khib  regulation |
| P08238 | 182 | K | HSP90AB1 | - | down |
| P08238 | 265 | K | HSP90AB1 | - | down |
| P08238 | 557 | K | HSP90AB1 | up | down |
| P08238 | 646 | K | HSP90AB1 | up | - |
| P08238 | 649 | K | HSP90AB1 | up | - |

| Table S6 Kcr and Khib sites on HSPD1 in this study | | | | | |
| --- | --- | --- | --- | --- | --- |
| Protein accession | Position | Amino  acid | Gene  name | Kcr  regulation | Khib  regulation |
| P10809 | 191 | K | HSPD1 | - | up |
| P10809 | 249 | K | HSPD1 | - | up |
| P10809 | 364 | K | HSPD1 | - | down |
| P10809 | 417 | K | HSPD1 | up | - |

| Table S7 Kcr and Khib sites on PDIA3 in this study | | | | | |
| --- | --- | --- | --- | --- | --- |
| Protein accession | Position | Amino  acid | Gene  name | Kcr  regulation | Khib  regulation |
| P30101 | 233 | K | PDIA3 | - | up |
| P30101 | 288 | K | PDIA3 | down | - |
| P30101 | 296 | K | PDIA3 | - | up |

| Table S8 Kcr and Khib sites on CLTC in this study | | | | | |
| --- | --- | --- | --- | --- | --- |
| Protein accession | Position | Amino  acid | Gene  name | Kcr  regulation | Khib  regulation |
| Q00610 | 619 | K | CLTC | up | down |

| Table S9 Kcr and Khib sites on MSN in this study | | | | | |
| --- | --- | --- | --- | --- | --- |
| Proein  accession | Position | Amino  acid | Gene  name | Kcr  regulation | Khib  regulation |
| P26038 | 72 | K | MSN | - | up |
| P26038 | 79 | K | MSN | down | up |
| P26038 | 83 | K | MSN | - | up |
| P26038 | 151 | K | MSN | - | up |
| P26038 | 263 | K | MSN | - | up |
| P26038 | 316 | K | MSN | up | - |
| P26038 | 335 | K | MSN | up | - |
| P26038 | 352 | K | MSN | up | - |
| P26038 | 388 | K | MSN | up | - |
| P26038 | 400 | K | MSN | up | - |
| P26038 | 458 | K | MSN | up | - |
| P26038 | 514 | K | MSN | up | - |
| P26038 | 523 | K | MSN | - | up |

| Table S10 Kcr and Khib sites on ACTN4 in this study | | | | | |
| --- | --- | --- | --- | --- | --- |
| Protein accession | Position | Amino  acid | Gene  name | Kcr  regulation | Khib  regulation |
| O43707 | 122 | K | ACTN4 | down | - |
| O43707 | 437 | K | ACTN4 | up | - |
| O43707 | 450 | K | ACTN4 | - | up |
| O43707 | 518 | K | ACTN4 | - | up |
| O43707 | 592 | K | ACTN4 | - | up |
| O43707 | 625 | K | ACTN4 | down | - |

| Table S11 Kcr and Khib sites on ACTN1 in this study | | | | | |
| --- | --- | --- | --- | --- | --- |
| Protein accession | Position | Amino  acid | Gene  name | Kcr  regulation | Khib  regulation |
| P12814 | 398 | K | ACTN1 | down | up |
| P12814 | 436 | K | ACTN1 | up | - |
| P12814 | 95 | K | ACTN1 | down | - |
| P12814 | 195 | K | ACTN1 | up | - |

| Table S12 Kcr and Khib sites on RAC1 in this study | | | | | |
| --- | --- | --- | --- | --- | --- |
| Protein accession | Position | Amino  acid | Gene  name | Kcr  regulation | Khib  regulation |
| P63000 | 123 | K | RAC1 | up | - |
| P63000 | 128 | K | RAC1 | up | - |
| P63000 | 133 | K | RAC1 | - | up |
| P63000 | 153 | K | RAC1 | - | up |

| Table S13 Kcr and Khib sites on EZR in this study | | | | | |
| --- | --- | --- | --- | --- | --- |
| Protein accession | Position | Amino  acid | Gene  name | Kcr  regulation | Khib  regulation |
| P15311 | 64 | K | EZR | - | down |
| P15311 | 327 | K | EZR | - | down |
| P15311 | 344 | K | EZR | down | - |
| P15311 | 438 | K | EZR | - | up |

| Table S14 Kcr and Khib sites on VCL in this study | | | | | |
| --- | --- | --- | --- | --- | --- |
| Protein accession | Position | Amino  acid | Gene  name | Kcr  regulation | Khib  regulation |
| P18206 | 170 | K | VCL | down | - |
| P18206 | 352 | K | VCL | - | up |
| P18206 | 768 | K | VCL | - | down |
| P18206 | 1024 | K | VCL | up | - |

| Table S15 Kcr and Khib sites on RHOA in this study | | | | | |
| --- | --- | --- | --- | --- | --- |
| Protein accession | Position | Amino  acid | Gene  name | Kcr  regulation | Khib  regulation |
| P61586 | 135 | K | RHOA | down | - |
| P61586 | 140 | K | RHOA | - | up |
